# (-)-Leucophyllone, a Tirucallane Triterpenoid from *Cornus walteri*, Enhances Insulin Secretion in INS-1 Cells

**DOI:** 10.3390/plants10030431

**Published:** 2021-02-24

**Authors:** Dahae Lee, Ki Hyun Kim, Taesu Jang, Ki Sung Kang

**Affiliations:** 1 College of Korean Medicine, Gachon University, Seongnam 13120, Korea; pjsldh@gachon.ac.kr; 2 School of Pharmacy, Sungkyunkwan University, Suwon 16419, Korea; khkim83@skku.edu; 3 College of Medicine, Dankook University, Cheonan 31116, Korea

**Keywords:** *Cornus walteri*, cornaceae, (-)-leucophyllone, glucose-stimulated insulin secretion, PDX-1

## Abstract

Phytochemical examination of the MeOH extract from the stems and stem bark of *Cornus walteri* (Cornaceae) led to the isolation and verification of a tirucallane triterpenoid, (-)-leucophyllone, as a major component. Its structure was elucidated using NMR spectroscopy and liquid chromatography–mass spectrometry. The effect of (-)-leucophyllone on insulin secretion in INS-1 cells was investigated. (-)-Leucophyllone increased glucose-stimulated insulin secretion (GSIS) at concentrations showing no cytotoxic effect in rat INS-1 pancreatic β-cells. Moreover, we attempted to determine the mechanism of action of (-)-leucophyllone in the activation of insulin receptor substrate-2 (IRS-2), phosphatidylinositol 3-kinase (PI3K), Akt, and pancreatic and duodenal homeobox-1 (PDX-1). Treatment of INS-1 cells with (-)-leucophyllone markedly increased the expression of these proteins. Our findings indicate the potential of (-)-leucophyllone as an antidiabetic agent.

## 1. Introduction

Type 2 diabetes (T2D) is a chronic metabolic disorder primarily characterized by reduced insulin secretion, and its global incidence has been on the rise [1]. Glucose-stimulated insulin secretion (GSIS) is generally accepted as the main mechanism of insulin secretion [2]. Insufficient insulin secretion is a known defect in pancreatic β-cell function [3]. Thus, maintaining or enhancing pancreatic β-cell function could be a strategic approach for the prevention and treatment of T2D.

Several natural products isolated from plants, on account of their complex chemical structures, are known for their biological potency as drugs [4]. In particular, compounds isolated from various plants have been reported to enhance insulin secretion via the regulation of pancreatic β-cell function, including cytopiloyne from *Bidens pilosa* [5], capsaicin from *Capsicum annuum* [6], berberine from *Rhizoma coptidis* [7], curcumin from *Curcuma longa* [8], epigallocatechin-3-gallate from *Camellia sinensis* [9], and genistein from *Glycine max* [10]. However, very little is known about their possible mechanisms of action.

*Cornus walteri* Wanger, better known as Walter’s dogwood, is a deciduous shrub belonging to the family Cornaceae. This medicinal plant has received attention for its anti-photoaging and anti-diarrheal effects [11,12]. In addition, its extracts possess anti-hyperglycemic [13], anti-inflammatory [14], anti-oxidant, and anti-obesity effects [15]. This bioactive potential of *C. walteri* necessitates its detailed phytochemical investigation. Our previous phytochemical analysis on *C. walteri* led to the isolation of triterpenoids, including betulinic acid methyl ester, lupenone, lupeol, betulinic acid, methyl 3-*O*-acetyl betulinate, and sterols such as 6β-hydroxysitostenone, 5α-stigmast-3,6-dione, sitostenone, 3β-sitostanol, and 6α-hydroxy-β-sitostenone [16,17]. Among them, the anticancer effect of betulinic acid on A2780 human ovarian cancer cells and the renoprotective effect of benzyl salicylate on cisplatin-induced damage in LLC-PK1 kidney proximal tubule cells have been explored and evaluated in our previous studies [16,17]. In the present study, we focused on (-)-leucophyllone, a tirucallane triterpenoid isolated from the further phytochemical investigation of the MeOH extract from *C. walteri*. To the best of our knowledge, the biological activity of (-)-leucophyllone has not been reported. Therefore, we explored the insulin enhancement effect of (-)-leucophyllone in rat INS-1 pancreatic β-cells. Additionally, we studied the mechanism of action of (-)-leucophyllone in the activation of insulin receptor substrate-2 (IRS-2), phosphatidylinositol 3-kinase (PI3K), Akt, and pancreatic and duodenal homeobox-1 (PDX-1).

## 2. Results

### 2.1. Isolation and Identification of (-)-Leucophyllone from C. walteri

**Figure 1:**
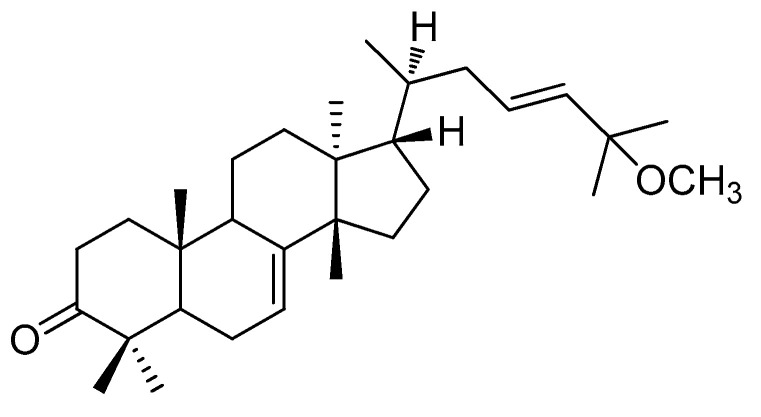
Chemical structure of (-)-leucophyllone.

Phytochemical analysis of the MeOH extract from the stems and stem bark of *C. walteri*, using successive column chromatography on silica gel and RP-C_18_ silica along with semi-preparative HPLC, resulted in the isolation and verification of (-)-leucophyllone from the hexane-soluble fraction. (-)-Leucophyllone was isolated as a white amorphous powder, and its molecular formula of C_31_H_50_O_2_ was determined based on LC/MS data at *m/z* 477.7 [M^+^ Na]^+^. The ^1^H (Figure S1) and ^13^C NMR (Figure S2) data of (-)-leucophyllone suggested the characteristic NMR spectroscopic values for eight methyl groups, one *trans*-disubstituted olefinic bond, one trisubstituted olefinic bond, one methoxy group, and one ketone group at δ_C_ 216.9, which were identical to the values of (-)-leucophyllone, an analogue of tirucallane-type triterpenoids reported from *Aglaia leucophylla* [18]. By comparing its negative value of optical specific data with previously reported data of (-)-leucophyllone [18], the absolute configuration of (-)-leucophyllone was determined as shown in Figure 1. Accordingly, the structural elucidation of (-)-leucophyllone isolated was unambiguously confirmed (Figure 1).

### 2.2. Glucose-Stimulated Insulin Secretion (GSIS) Effect

**Figure 2:**
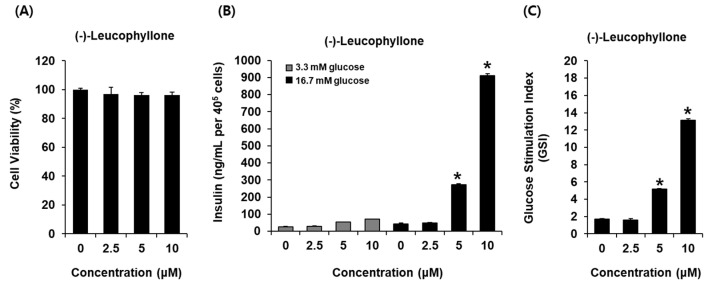
Effect of (-)-leucophyllone on glucose-induced insulin secretion in INS-1 cells. (**A**) Effect of (-)-leucophyllone on the viability of INS-1 cells following 24 h of incubation, compared to that of the control (0 μM), as determined by cell viability assay. (**B**) Effect of (-)-leucophyllone on glucose-stimulated insulin secretion (glucose-stimulated insulin secretion (GSIS), ng/mL per 400,000 cells) in INS-1 cells following 1 h of treatment, compared to that of the control (0 μM), as determined using the GSIS assay. (**C**) Comparison of GSIS is expressed as fold-stimulation in terms of the glucose-stimulated index (GSI, 16.7 mM glucose over 2.8 mM glucose for 1h). *n* = 3 independent experiments, * *p* < 0.05, Kruskal–Wallis non-parametric test. The data are presented as the mean ± SEM.

In this study, we investigated whether (-)-leucophyllone could enhance GSIS and if it was cytotoxic to INS-1 cells. (-)-Leucophyllone at 2.5 μM, 5 μM, and 10 μM was found to be nontoxic to INS-1 cells (Figure 2A). The non-toxic concentrations of (-)-leucophyllone were tested to determine if they led to an increase in GSIS. As shown in Figure 2B, (-)-leucophyllone increased GSIS (ng/mL per 400,000 cells). GSIS was expressed as the glucose-stimulated index (GSI). Fold change was set at 1 for control. The resultant GSI values were found to be 5.16 ± 0.12 and 13.11 ± 0.17 for (-)-leucophyllone at 5 μM and 10 μM, respectively (Figure 2C). The results suggested that (-)-leucophyllone enhanced insulin secretion in response to high glucose without causing exhibiting toxicity to INS-1 cells.

### 2.3. Protein Expression of IRS-2 (Ser731), P-IRS-2, PI3K, P-PI3K, Akt, P-Akt (Ser473), and PDX-1

**Figure 3:**
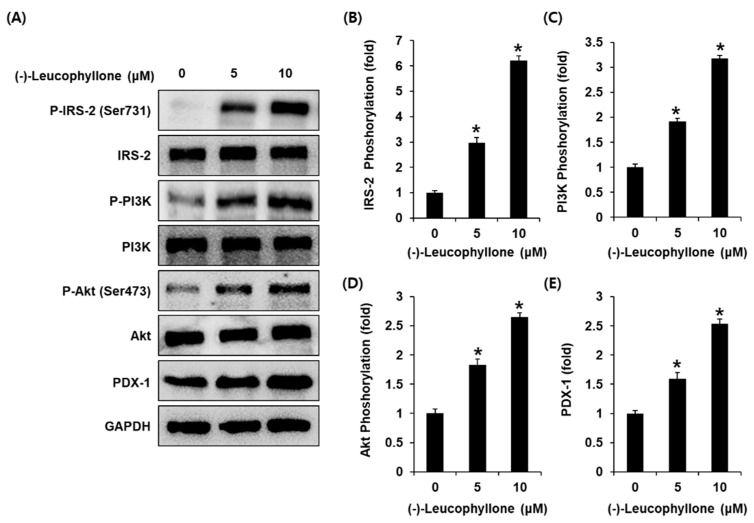
Effect of (-)-leucophyllone on the protein expression levels of insulin receptor substrate-2 (IRS-2) (Ser731), P-IRS-2, P-phosphatidylinositol 3-kinase (PI3K), PI3K, P-Akt (Ser473), Akt, and pancreatic and duodenal homeobox-1 (PDX-1) in INS-1 cells. (**A**) Protein expression levels of P-IRS-2 (Ser731), IRS-2, P-PI3K, PI3K, P-Akt (Ser473), Akt, PDX-1, and glyceraldehyde 3-phosphate dehydrogenase (GAPDH) in INS-1 cells treated or untreated with 5 and 10 μM (-)-leucophyllone for 24 h. (**B**–**E**) Each bar graph presents the densitometric quantification of western blot bands. * *p* < 0.05 compared to the control (0 μM).

**Figure 4:**
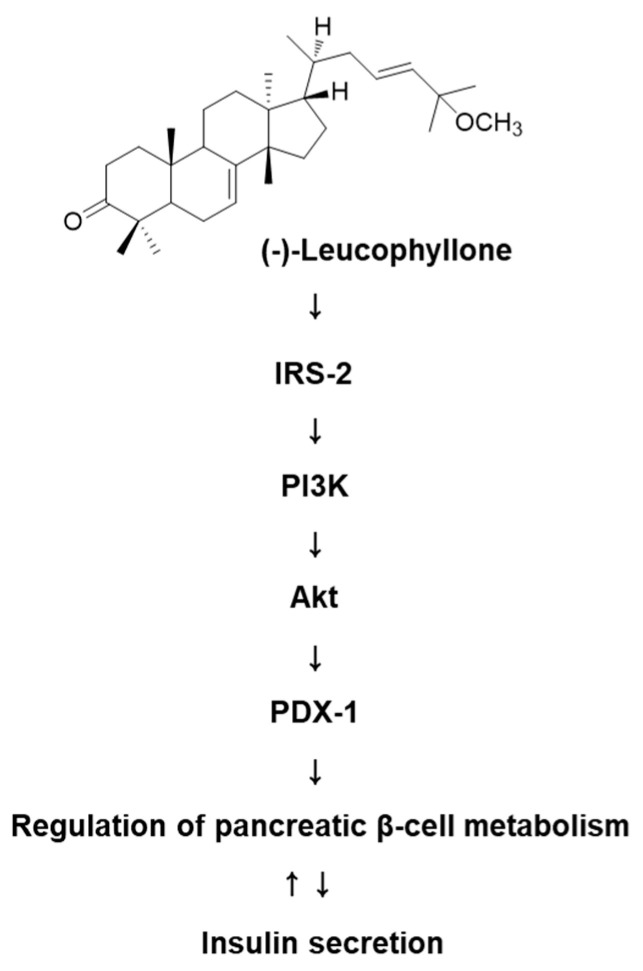
Schematic illustration of the effect of (-)-leucophyllone isolated from *C. walteri* on the protein expression levels of insulin receptor substrate-2 (IRS-2), phosphatidylinositol 3-kinase (PI3K), Akt, and pancreatic and duodenal homeobox-1 (PDX-1) in INS-1 cells.

Treatment with (-)-leucophyllone at 5 μM and 10 μM increased the protein expression of IRS-2, PI3K, Akt, and PDX-1 compared to the untreated controls in INS-1 cells (Figure 3). These results suggested that (-)-leucophyllone upregulated PDX-1 expression via the IRS-2/PI3K/Akt signaling pathway in INS-1 cells. A schematic illustration of the proposed mechanisms of the effect of (-)-leucophyllone on pancreatic β-cell metabolism is shown in Figure 4.

## 3. Discussion

Insulin is the only hormone responsible for lowering plasma glucose levels. Glucose homeostasis is maintained when the insulin secretion is normal [19]. Because GSIS is a vital concept in the development of T2D, it needs to be explored as a strategy to discover novel bioactive compounds to treat T2D [20]. In the present study, we investigated whether (-)-leucophyllone could enhance GSIS. Moreover, we evaluated its cytotoxicity in INS-1 cells. Based on our results, we concluded that while (-)-leucophyllone did not increase cell viability; it also did not induce cytotoxicity at any concentration that was used in our study. In addition, we found that (-)-leucophyllone enhanced insulin secretion in response to high glucose levels in INS-1 cells. Therefore, we suggest that (-)-leucophyllone may enhance GSIS by modulating cellular signals rather than increasing the viability of insulin-secreting cells.

Flavonoids are known to upregulate protein expression, including the expression of IRS-2 and PDX-1, thereby enhancing insulin secretion in pancreatic β cells [21]. Phosphorylated IRS-2 has essential roles, including the regulation of normal pancreatic β-cell function, particularly, the maintenance of pancreatic β-cell mass and activation of the PI3K/Akt pathway [22,23]. Although its role is not fully understood, the PI3K/Akt pathway is critical for the nuclear translocation of PDX-1 [24,25]. PDX-1 is a crucial transcription factor that maintains pancreatic β-cell function in normal GSIS and activates the insulin gene promoter [26,27]. Its deficiency has been shown to cause defective GSIS in mouse and human pancreatic β cells [26,28,29]. The polyphenol extract of *Caesalpinia bonduc* was previously shown to enhance insulin secretion and PDX-1 expression in pancreatic β cells of rats [30]. It has been demonstrated that the extract of mistletoe (*Viscum album coloratum*) has an insulin-secreting effect through the activation of PDX-1 in alloxan-induced diabetic mice [31]. Another study showed that the polysaccharide extracted from mulberry (*Morus alba* L.) leaf ameliorated insulin-secreting activity by increasing PDX-1 expression through nuclear localization in the pancreatic β cells of diabetic rats [32].

In our study, we also investigated the involvement of the pathway associated with β-cell function in INS-1 cells after treatment with (-)-leucophyllone. Our results were consistent with the theory suggested by previous studies that PDX-1 expression via the IRS-2/PI3K/Akt signaling pathway played a key role in insulin secretory capacity. In the current study, treatment with (-)-leucophyllone increased the protein expression of IRS-2, PI3K, Akt, and PDX-1 compared to untreated controls in INS-1 cells. These results established the underlying mechanism of action of (-)-leucophyllone to enhance GSIS. However, the biggest drawback of natural product research is still difficult to separate enough components for animal testing, and we need to further study the mechanism of action using other experimental models and diabetic animals in future studies.

## 4. Materials and Methods

### 4.1. Extraction, Fractionation, and Purification Methods

*Cornus walteri* stems and stem bark (2.5 kg) were dried, chopped, and extracted with 80% aqueous MeOH (2 × 6 h) under reflux, and the resultant extract was filtered. The filtrate was concentrated using evaporator under vacuum to afford a MeOH extract (220 g), which was applied to distilled H_2_O (7.2 L) and then successively solvent-partitioned with three organic solvents including hexane, CHCl_3_, and *n*-BuOH, providing 9.5, 25, and 43 g of residue, respectively. The hexane-soluble fraction (9.5 g) was chromatographed on a 300 g of silica gel column eluted with hexane-EtOAc (3:1 to 1:1, gradient solvent system) to yield fractions F1–F5. Fraction F1 (3.3 g) was chromatographed on a 100 g of RP-C18 silica gel column eluted with 100% MeOH to yield subfractions F11–F15. Fraction F14 (300 mg) was subjected to medium pressure liquid chromatography (MPLC) using a LiChroprep Lobar-A Si gel 60 column (*n*-hexane-EtOAc, 16:1) and then purified using semi-preparative normal-phase HPLC and a solvent system comprising *n*-hexane:EtOAc (12:1) to yield (-)-leucophyllone (30 mg, 0.0136%).

### 4.2. Cell Culture

Rat pancreatic β-cells (INS-1) were purchased from Biohermes (Shanghai, China). INS-1 cells were routinely maintained in RPMI-1640 (Cellgro, Manassas, VA, USA) containing 11 mM d-glucose, 10% fetal bovine serum, 1% penicillin/streptomycin (Invitrogen Co., Grand Island, NY, USA), 0.05 mM 2-mercaptoethanol, 2 mM l-glutamine, 10 mM HEPES, and 1 mM sodium pyruvate under 5% CO_2_ and 95% humidity at 37 °C.

### 4.3. Measurement of Cell Viability

To assess the non-toxic dose range of (-)-leucophyllone, INS-1 cells (1 × 10^4^ cells/well) were seeded in 96-well plates for 24 h. Cells were then treated with 2.5, 5, or 10 μM (-)-leucophyllone for 24 h. EZ-Cytox cell viability assay solution (100 μL; Daeil Lab Service Co., Seoul, Korea) was added to the plates and incubated for 40 min. Next, the absorbance of the samples in the wells was measured using a PowerWave XS microplate reader (Bio-Tek Instruments, Winooski, VT, USA) at a wavelength of 450 nm as previously described [33,34].

### 4.4. GSIS Assay

INS-1 cells (4 × 10^5^ cells/well) were seeded in 12-well plates for 24 h. Cells were then carefully washed twice with warm Krebs–Ringer bicarbonate buffer (KRBB, 4.8 mM KCl, 129 mM NaCl, 1.2 mM KH_2_PO_4_, 1.2 mM MgSO_4_, 2.5 mM CaCl_2_, 10 mM HEPES, 5 mM NaHCO_3_, and 0.1% bovine serum albumin (pH 7.4). Then, INS-1 cells were starved with fresh KRBB for 2 h and treated with 2.5, 5, and 10 μM (-)-leucophyllone for 1 h. Thereafter, INS-1 cells were stimulated with fresh KRBB containing 2.8 mM or 16.7 mM glucose for 1 h. The culture supernatant was immediately collected and used to measure the GSIS according to the manufacturer’s instructions for the rat insulin ELISA kit (Gentaur, Shibayagi Co. Ltd., Gunma, Shibukaw, Japan). GSIS is expressed as fold-stimulation in terms of the glucose-stimulated index (16.7 mM glucose over 2.8 mM glucose for 1 h).

### 4.5. Western Blotting

INS-1 cells (8 × 10^5^ cells/well) were seeded in six-well plates for 24 h. Cells were then treated with 5 or 10 μM (-)-leucophyllone for 24 h. To extract the whole protein lysate, INS-1 cells were lysed for 20 min on ice in RIPA buffer (Cell Signaling, Danvers, MA, USA) containing a protease inhibitor. Protein samples (20 μg) were separated and detected as previously described [35,36].

### 4.6. Statistical Analysis

Statistical significance was assessed using one-way analysis of variance (ANOVA) and multiple comparisons with a Bonferroni correction. *p* values less than 0.05 indicated statistical significance. All analyses were performed using SPSS Statistics ver. 19.0 (SPSS Inc., Chicago, IL, USA).

## 5. Conclusions

Based on the results from our study, we conclude that (-)-leucophyllone identified from *C. walteri* was capable of GSIS. These effects were supported by the increased expression of IRS-2, PI3K, Akt, and PDX-1. Further studies are necessary to investigate the impact of (-)-leucophyllone on insulin secretion in animal models of T2D and to evaluate whether (-)-leucophyllone might be of therapeutic interest for the treatment of T2D in humans.

## Data Availability

Not applicable.

## Supplementary Materials

The following are available online at https://www.mdpi.com/2223-7747/10/3/431/s1, general experimental procedures, plant material, Figure S1: The ^1^H NMR spectrum of (-)-leucophyllone (CDCl_3_, 500 MHz); Figure S2: The ^13^C NMR spectrum of (-)-leucophyllone (CDCl_3_, 125 MHz).
