# Supplementary material for: (-)-Leucophyllone, a Tirucallane Triterpenoid from Cornus walteri, Enhances Insulin Secretion in INS-1 Cells
Source: Plants (Basel). 2021 Feb 24;10(3):431. doi: 10.3390/plants10030431 (PMC7996230; doi:10.3390/plants10030431)

Supplementary Material

(-)-Leucophyllone, a Tirucallane Triterpenoid from *Cornus walteri*, Enhances Insulin Secretion in INS-1 Cells

Dahae Lee [1], Ki Hyun Kim [2], Taesu Jang [3,*] and Ki Sung Kang [1,*]

[1] College of Korean Medicine, Gachon University, Seongnam 13120, Korea; pjsldh@gachon.ac.kr
[2] School of Pharmacy, Sungkyunkwan University, Suwon 16419, Korea; khkim83@skku.edu
[3] College of Medicine, Dankook University, Cheonan 31116, Korea
* Correspondence: jangts@dankook.ac.kr (T.J.); kkang@gachon.ac.kr (K.S.K.); Tel.: +82-41-550-1476 (T.J.); +82-31-750-5402 (K.S.K.)

General experimental procedures

Optical rotation was measured using a Jasco P-1020 polarimeter and methanol as a solvent. IR spectra were recorded using a Bruker IFS-66/S FT-IR spectrometer and methanol as a solvent. Ultraviolet (UV) spectra were recorded using Shimadzu UV-1601 UV-Visible spectrophotometer and using methanol as a solvent. Electrospray ionization (ESI) MS spectra were recorded on a Micromass QTOF2-MS. LC/MS analysis was performed using an Agilent 1200 Series HPLC system equipped with a diode array detector and a 6130 Series ESI mass spectrometer using an analytical Kinetex (2.1 × 100 mm, 5 μm). NMR spectra were recorded using a Varian UNITY INOVA 500 NMR spectrometer operating at 500 MHz (^{1}H) and 125 MHz (^{13}C), with chemical shifts given in ppm (δ). Preparative HPLC was performed using a Gilson 306 pump with a Shodex refractive index detector. Silica gel 60 (Merck, 230-400 mesh) and RP-C18 silica gel (Merck, 230-400 mesh) were used for column chromatography. Merck precoated silica gel F254 plates and RP-18 F254s plates were used for thin-layer chromatography (TLC). Spots were detected on TLC under UV light or by heating after spraying with anisaldehyde-sulfuric acid.

Plant material

Stems and stem bark of *C. walteri* were collected from Jeju Island, Korea in October 2005, and the plant was identified by one of the authors (K. H. Kim). A voucher specimen (SKKU 2005-10a) was deposited in the herbarium of the School of Pharmacy, Sungkyunkwan University, Suwon, Korea.

Figure 1. The ^{1}H NMR spectrum of (-)-leucophyllone ($CDCl_3$, 500 MHz).

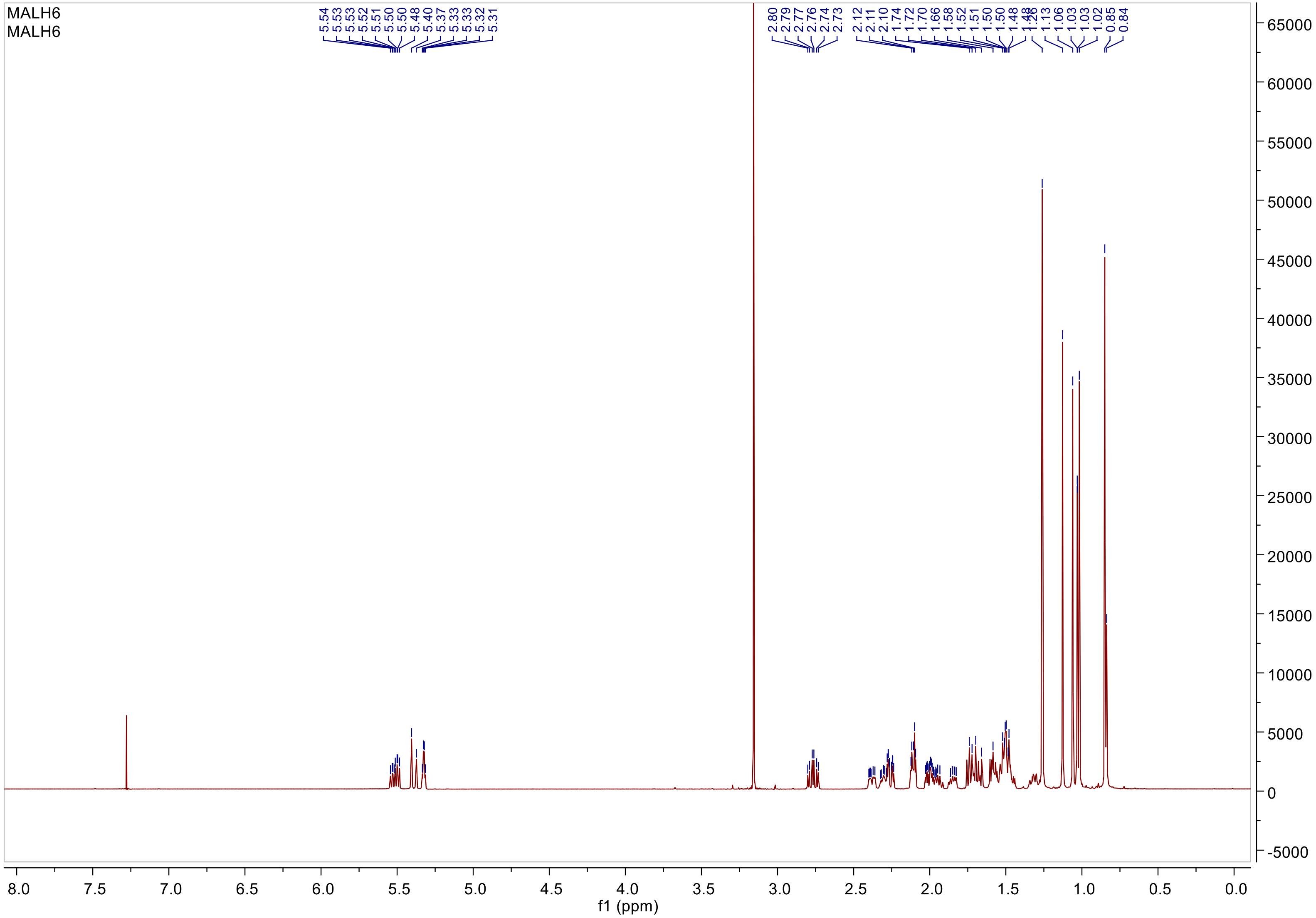

Figure 2. The ^{13}C NMR spectrum of (-)-leucophyllone ($CDCl_3$, 125 MHz).

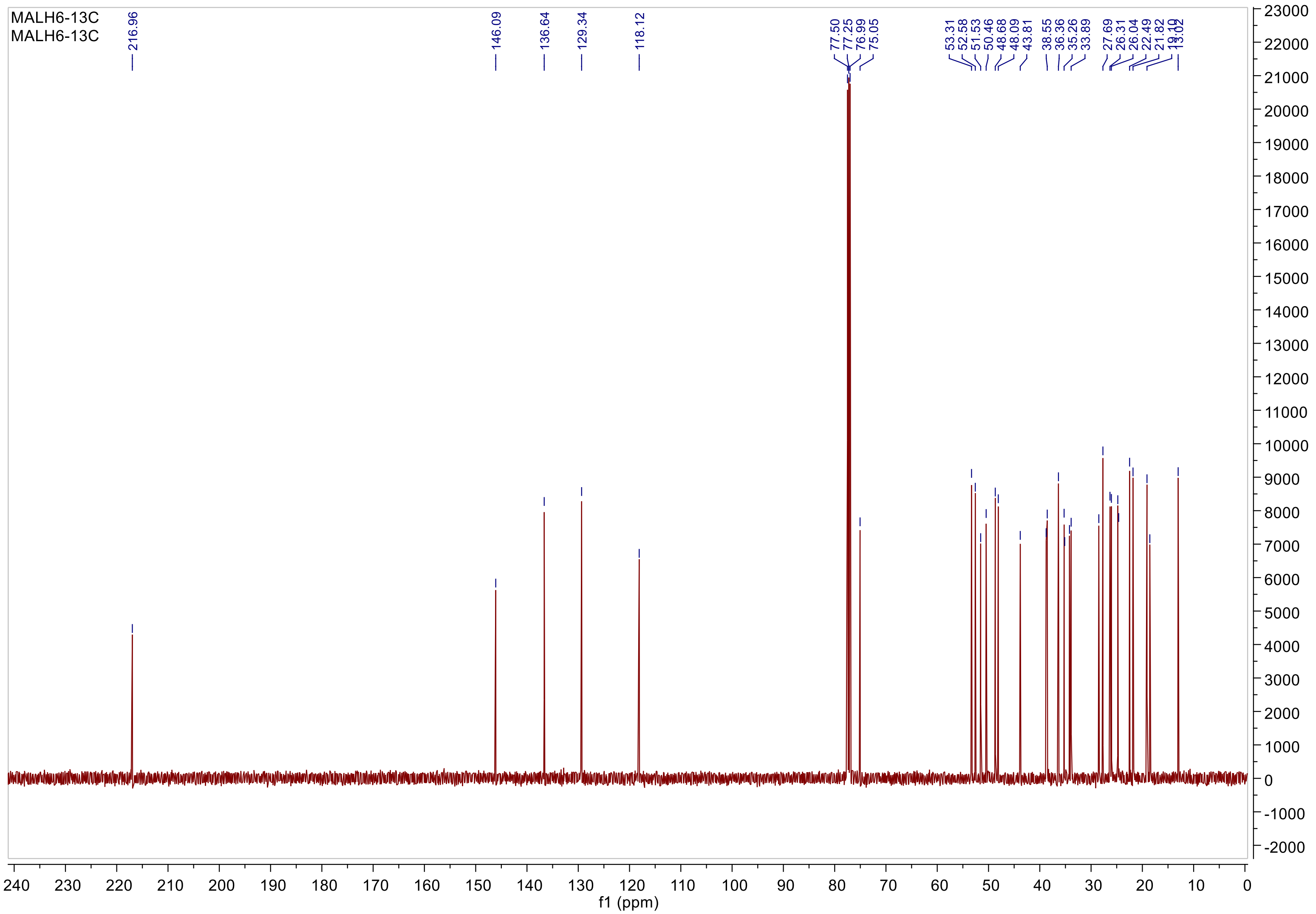

Supplement: Supplementary file 1 [file plants-10-00431-s001.pdf]
